# Molecular and antigenic characterization of group C orthobunyaviruses isolated in Peru

**DOI:** 10.1371/journal.pone.0200576

**Published:** 2018-07-19

**Authors:** Roger M. Castillo Oré, Roxana E. Caceda, Alfredo A. Huaman, Maya Williams, Jun Hang, Diana E. Juarez, Tadeusz J. Kochel, Eric S. Halsey, Brett M. Forshey

**Affiliations:** 1 U.S. Naval Medical Research Unit No. 6, Iquitos and Lima, Peru; 2 U.S. Naval Medical Research Center, Silver Spring, Maryland, United States of America; 3 Viral Diseases Branch, Walter Reed Army Institute of Research, Silver Spring, Maryland, United States of America; University of Texas Medical Branch at Galveston, UNITED STATES

## Abstract

Group C orthobunyaviruses (GRCVs) are a complex of viruses in the genus *Orthobunyavirus* and are associated with human febrile disease in tropical and subtropical areas of South and Central America. While numerous GRCVs have been isolated from mosquitoes, animals, and humans, genetic analysis of these viruses is limited. In this study, we characterized 65 GRCV isolates from febrile patients identified through clinic-based surveillance in the northern and southern Peruvian Amazon. A 500 base pair region of the S segment and 750 base pair regions of the M and L segments were sequenced. Pairwise sequence analysis of the clinical isolates showed nucleotide identities ranging from 68% to 100% and deduced amino acid sequence identities ranging from 72% to 100%. Sequences were compared with reference strains of the following GRCVs: Caraparu virus (CARV), Murutucu virus (MURV), Oriboca virus (ORIV), Marituba virus (MTBV), Itaqui virus (ITQV), Apeu virus (APEUV), and Madrid virus (MADV). Sequence comparison of clinical isolates with the prototype strains based on the S and L segments identified two clades; clade I included isolates with high genetic association with CARV-MADV, and clade II included isolates with high genetic association with MURV, ORIV, APEUV, and MTBV. Genetic relationships based on the M segment were at time inconsistent with those based on the S and L segments. However, clade groupings based on the M segment were highly consistent with relationships based on microneutralization assays. These results advance our understanding of the genetic and serologic relationships of GRCVs circulating in the Peruvian Amazon.

## Introduction

During 1954–1959, the *Servico Especial de Saude Publica* of Brazil and the Rockefeller Foundation conducted studies to isolate arboviruses in the Brazilian Amazon region and isolated a novel group of vector-borne viruses, designated as group C (GRCV). These isolates were grouped serologically as 6 viruses named Marituba, Oriboca, Apeu, Murutucu, Caraparu and Itaqui [1–4]. GRCVs are maintained in nature by cycles involving animals, including rodents, monkey, marsupials, bats and mosquitoes in tropical and subtropical areas of the Americas, and, as with other arboviruses, the geographic distributions are determined by their specific vector and host requirements [5–7]. Humans are considered accidental hosts, and infections caused by GRCV have been associated with sporadic, non-fatal disease causing high fever, headache, chills, myalgia, photophobia, and retroocular pain, usually lasting 4–5 days [2,5,8].

GRCV classification was initially based on antigenic relationships determined using complement fixation, neutralization, and hemagglutination inhibition tests. The earliest studies on the serological relationships showed broad cross reactions between prototypes, depending on the assays: hemagglutination inhibition and virus neutralization tests indicated similarities between Oriboca virus and Itaqui virus, Apeu virus and Caraparu virus, Murutrucu virus and Marituba virus, while complement fixation tests indicated similarities between Itaqui virus and Caraparu virus, Apeu virus and Marituba virus, Murutucu virus and Oriboca virus [3].

Currently GRCVs are grouped in the family *Peribunyaviridae*, genus *Orthobunyavirus* and represented in the International Committee on Taxonomy of Viruses (ICTV) database (https://talk.ictvonline.org/) by the following species: *Caraparu orthobunyavirus* (CARV), *Oriboca orthobunyavirus* (ORIV), *Madrid orthobunyavirus* (MADV) and *Marituba orthobunyavirus* (MTBV).

Like the other members of the genus *Orthobunyavirus*, the virions are spherical, enveloped in lipid, and possess an outer fringe of glycoprotein. The genome is composed of three segments of negative-sense, single-stranded RNA: large (L) ~ 7000 nucleotides (nt), medium (M) ~ 4500 nt, and small (S) ~ 1000 nt. The L segment encodes an internal large protein RNA-dependent RNA polymerase (RdRp). The M segment encodes the two external viral glycoproteins Gn and Gc, which are inserted in the viral membrane, and also additionally encodes a non-structural protein NSm. The S segment encodes two proteins in overlapping reading frames, the internal nucleocapsid (N) protein and a non-structural protein (NSs) [9–11].

In Peru, the first isolations of GRCV occurred in 1970 and 1971 from animal samples collected in the Amazon region [12]. More recently, GRCVs have also been recovered from febrile human samples in Peru [13]. Despite their established role in human disease, little is known about the serologic and molecular genetic characteristics of GRCVs circulating in Peru. The objective of this study was to describe the antigenic and genetic relationships of GRCVs isolated from human febrile cases in the Amazon basin of Peru.

## Materials and methods

### Study

Serum samples were collected from subjects participating in a passive clinic-based surveillance study of febrile disease between 1995 and 2013 [13,14], covered by protocols DoD 30517, NMRCD.2000.0006 and NMRCD.2010.0010. The first two protocols were approved by the institutional review board (IRB) at the Naval Medical Research Center (Silver Spring, MD) and the last one was approved by the IRB at Naval Medical Research Unit No. 6 (NAMRU-6; Lima, Peru). Additionally, the protocols were reviewed and approved by the Peruvian Ministry of Health. Surveillance occurred at outpatient medical clinics in the following Peruvian cities: Iquitos, Puerto Maldonado, Yurimaguas, Piura, Tumbes, Cusco, Ucayali, Lima and La Merced. Inclusion criteria included age ≥ 5 years, presence of fever 38°C occurring for ≤ 7 days, and no obvious source of infection, such as cellulitis, dental abscess, influenza-like illness, or urinary tract infection.

### Virus culture and RNA extraction

Serum samples from febrile patients were inoculated onto Vero cell cultures and identified as GRCVs using an immunofluorescent assay as previously described [13]. Six GRCV reference strains obtained from Dr. Robert Tesh and the World Reference Center for Emerging Viruses and Arboviruses at the University of Texas Medical Branch, Galveston, TX and 65 isolates (Table 1 and S1 Table) were propagated in Vero cells and harvested when 70–100% of cytopathic effect was exhibited. Infected supernatant were centrifuged at 2000 g at 4°C for 20 minutes, and the clarified supernatants were recovered and stored in cryovials at -70°C. Viral RNA was extracted from viral culture clarified supernatant using the QIAamp Viral RNA Kit (Qiagen, Valencia, CA).

**Table 1:** Group C orthobunyavirus (GRCV) reference strains used in the study.

Year	Strain	Isolation origin	Source (host)	GenBank accession numbers (Segments S, M and L)
1956	Caraparu (CARV) BeAn3994	Belem, Brazil	*Cebus apella* serum	MH052061, MH052128, MH052195
1954	Oriboca (ORIV) BeAn17	Belem, Brazil	*Cebus apella* serum	MH052062, MH052129, MH052196
1955	Murutucu (MURV) BeAn974	Belem, Brazil	*Cebus apella* serum	MH052063, MH052130, MH052197
1955	Apeu (APEUV) BeAn848	Belem, Brazil	*Cebus apella* serum	MH052064, MH052131, MH052198
1954	Marituba (MTBV) BeAn15	Belem, Brazil	*Cebus apella* serum	MH052065, MH052132, MH052199
1996	Itaqui (ITQV) PE00036	Loreto, Peru	Mosquito pool	MH052066, MH052133, MH052200

Reference group C viruses listed, were obtained from Dr. Robert Tesh and the World Reference Center for Emerging Viruses and Arboviruses at the University of Texas Medical Branch, Galveston, TX.

### Reverse transcription and cDNA amplification

More than 500 base pair region of the S segment (coding sequence of partial N and NSs protein) and more than 750 base pair regions of the M (coding sequence of partial polyprotein, corresponding to GC site) and L (coding sequence of partial RdRp) segments were amplified using one step reverse transcription and PCR (RT-PCR). Primers (Table 2) were designed based on previous published sequences [15,16].

**Table 2:** Primers used in RT-PCR and sequencing for group C orthobunyavirus (GRCV).

Primer name*	Nucleotide sequence (5´to 3´)
GCN6-S282-F	AGGCTAAACAGAARCTSCGTAAGAG
GCN6-S838-R	CATKGTRATKCCAAACTCCTTRA
GCN6-M3306-F	TGYAARGAYATAATHAARCCWGA
GCN6-M4108-R	TCRCTRTTNCCDACTTCRAT
GCN6-L3877-F	AGYYTRGCATGGGTVAGYATTGC
GCN6-L4685-R	AAATCTYTYTTDATAGCARYAAATGTTTCWGG

* The primer name reflects the genomic segment (S, M or L), number corresponding to nucleotide position of each primer, and primer orientation forward (F), reverse (R).

RT-PCR was performed in a total volume of 50 μl containing 1 μM of each primer (forward and reverse; Table 2), 200 μM of deoxynucleoside triphosphates (dNTPs) (Promega, Madison, WI), 5 μM dithiothreitol (DTT), 5 units of avian myeloblastosis virus reverse transcriptase (AMV RT; Promega, Madison, WI), 2.5 units of GoTaq DNA polymerase (Promega, Madison, WI), 1X green GoTaq reaction buffer (Promega, Madison, WI), and 10 μl of extracted RNA.

For the partial S segment, reverse transcription was performed for 60 minutes at 42°C and 95°C for 2 minutes, followed by 35 PCR cycles, consisting of 94°C for 40 seconds, 50°C for 40 seconds, and 72°C for 50 seconds, and finally at 72°C for 5 minutes.

For the partial M and L segments, reverse transcription was performed for 60 minutes at 42°C and 95°C for 2 minutes, followed by 40 PCR cycles, consisting of 94°C for 40 seconds, 45°C for 40 seconds, and 72°C for 1 minutes, and finally at 72°C per 10 minutes. The amplified products were visualized under UV light after electrophoresis in a 2.0% agarose gel for the S segment and a 1.5% agarose gel for the M and L segments.

### Nucleotide sequencing

The cDNA amplicons from the partial S segment were purified using centri-sep columns (Princeton Separations, Inc.) after a single band was apparent in the gel. The amplicons from the partial M and L segments were recovered from excised DNA bands from the agarose gel and purified using Wizard SV Gel and PCR Clean-Up System (Promega, Madison, WI, USA). Sequencing was performed using BigDye® Terminator v3.1 Cycle Sequencing Kit (Applied Biosystems) and the same primers as used for RT-PCR. Sequencing reactions were purified using centri-sep columns (Princeton Separations, Inc.) or an ethanol precipitation method. Nucleotide sequences were determined by running purified sequencing reactions on the ABI 3130 XL Genetic Analyzer (Applied Biosystems).

### Sequence analysis and phylogeny

Nucleotide sequences were assembled using Sequencher 4.8 software (Gene Codes Corporation, Ann Arbor, MI). In addition to the sequences that were generated for this study (65 isolates and 6 reference strains), the following sequences from GenBank were included in the genetic analysis: Madrid Virus—BT4075 (GenBank accession numbers KF254781, KF254780 and KF254779) [16] and IQT9646 (GenBank accession numbers KM092514, KM092513, KM092512) [17].

ClustalW multiple alignment, pairwise comparisons, multiple sequence analyses and phylogenetic tree construction were performed using BioEdit 7.2 [18] and MEGA 5.2 [19] software. Trees were constructed using the neighbor-joining method and distances were calculated using Kimura 2-parameter model with bootstrap analysis using 1000 replicates.

### Neutralization test

Hyperimmune mouse ascitic fluid (HMAF) against APEUV BeAn848, CARV BeAn3994, ITQV (isolate PE-00036, collected from a mosquito pool in Peru [20]), MTBV BeAn15, MURV BeAn974 and ORIV BeAn17 was prepared by serial immunization of 6–8 week old BALB/c female mice as previously described [21,22]. The six HMAFs produced (HMAF-APEUV, HMAF-CARV, HMAF-ITQV (PE00036), HMAF-MTBV, HMAF-MURV and HMAF-ORIV) were heat inactivated at 56°C for 30 minutes, and using 96 well plates, two fold serial dilutions were made (from 1/20 to 1/2560) using cell culture media (50 μl of final volume for each dilution). Equal volumes of the heat inactivated serially diluted HMAF and each previously diluted virus isolate was mixed and incubated for 1 hour at 37°C to allow neutralization. The virus dilution used was determined by a preliminary titration, which contained between 30 and 300 TCID_50_, where one TCID_50_ is the dilution of the original stock which results in half the wells being infected and half the wells remaining uninfected, calculated using the method of Reed and Muench [23]. After incubation, virus and HMAF were mixed with 100 μl of Vero 76 cells at a concentration of 5 X 10^5^ / ml in cell culture media with 4% Fetal Bovine serum (FBS). The plates were incubated at 37°C in 5% CO_2_. After 3 days, supernatant media were removed and the cells were fixed and stained with a solution of naphthol blue black and acetic acid [24]. The neutralization titer of test sample was reported as the last dilution which cells are protected from cytopathic effect (last well in which at least 50% of the monolayer was protected, as identified by visual inspection of the monolayer).

## Results

**Fig 1:**
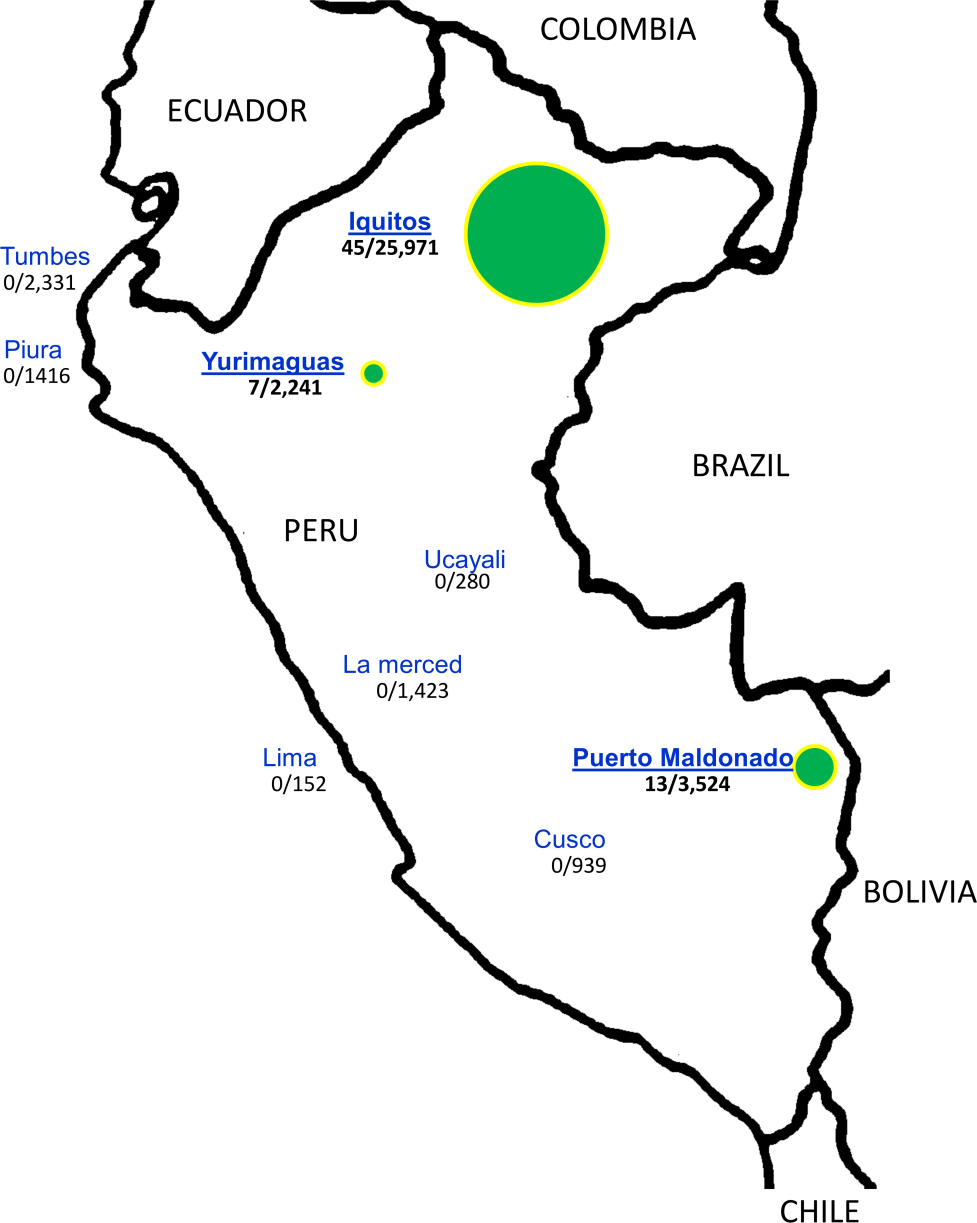
Distribution of febrile patient samples in Peru. Number of febrile surveillance samples (denominator) and number of group C isolates (numerator). The green circles represent the places where group C viruses were isolated and the size of the circle is proportional to the number of isolates.

In total, 65 GRCV isolates were identified between 1995 and 2013 from acute phase serum samples from febrile patients (S1 Table). GRCVs were isolated from patient samples collected in Iquitos (Northeastern Peru; 45 isolates from 25,293 acute phase samples tested), Yurimaguas (Northeastern Peru; 7 isolates from 2,241 samples), and Puerto Maldonado (Southeastern Peru; 13 isolates from 3,524 samples). In contrast, no GRCVs were isolated from Tumbes (0/2,331 acute phase samples tested), Piura (0/1,416), Ucayali (0/280), La Merced (0/1,423), Lima (0/152), or Cusco (0/939) (Fig 1). The median age of GRCV isolate-positive patients was 25 years (range 6–63 years); 62% were male (40/65; S1 Table). Infections with GRCVs commonly presented as an undifferentiated febrile illness, the most common symptoms described in the 65 patients were malaise (98%), headache (97%), chills (91%), arthralgia (84%), myalgia (84%), retro-ocular pain (83%), bone pain (63%), asthenia (51%), conjunctival injection (35%), pallor (32%), cough (19%), pharyngitis (16%), rash (14%), and expectoration (10%). Gastrointestinal manifestations occurred frequently, in particular decreased appetite (66%), nausea (54%), abdominal pain (41%), and vomiting (14%).

### S segment sequence analysis

**Fig 2:**
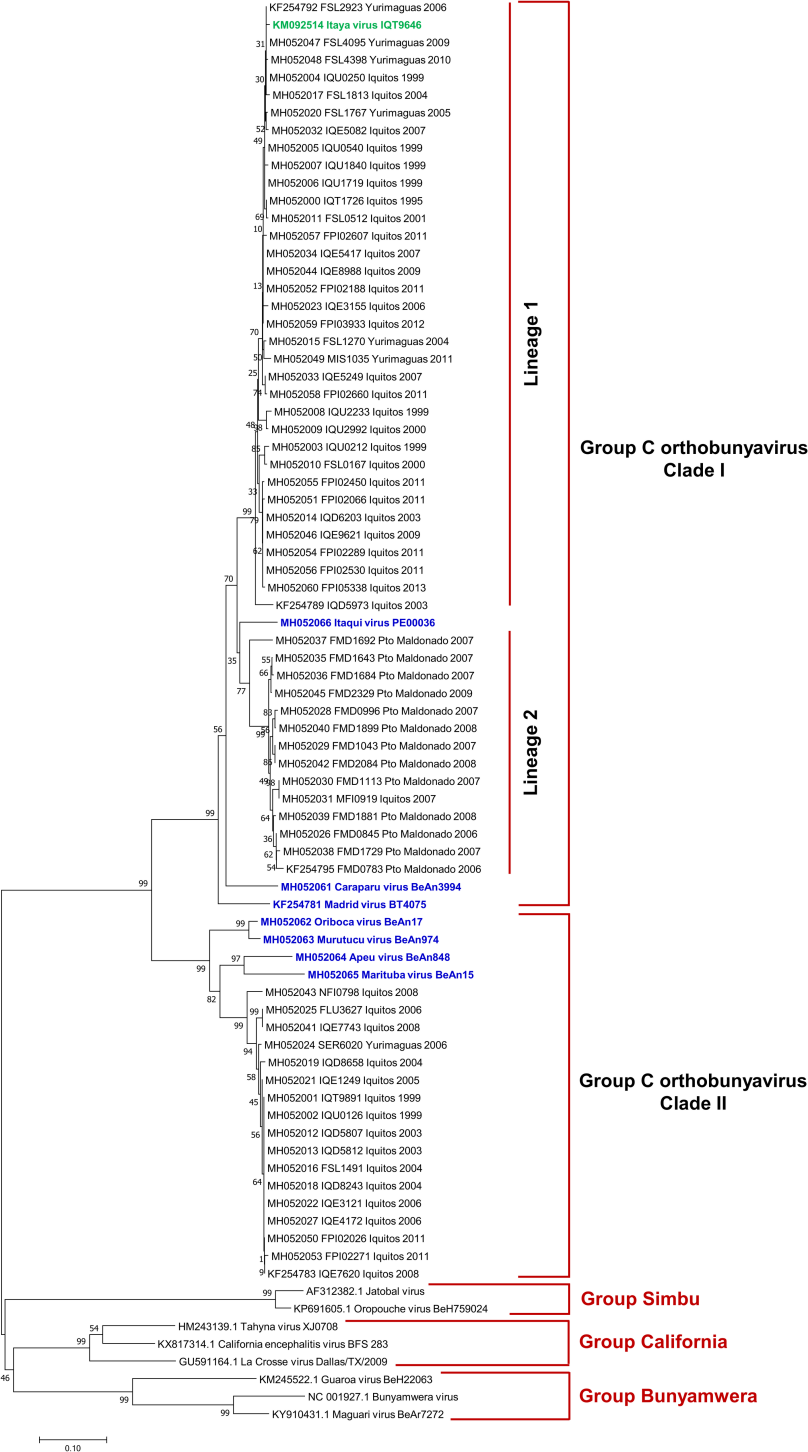
Phylogenetic analysis of group C viruses based on partial nucleotide sequences of the S segment. The phylogenetic tree was constructed using the neighbor-joining method. Bootstrap values were obtained based on 1000 replicates on the bases of partial coding sequence of N and NSs protein. The evolutionary distances were computed using the Kimura 2-parameter method. Members of the Simbu, California and Bunyamwera serogroups were used as outgroups to root the tree.

Based on partial S segment sequence, GRCV isolates grouped into two main clades (Fig 2). Pairwise comparisons between isolates from clade I and clade II ranged from 69% - 75% nucleotide identity and 75% - 77% amino acid identity (Table 3). Clade I comprised CARV, MADV, ITQV, and 49 clinical isolates (including one previously described isolate [17]). Clade I clinical isolates shared 86% - 88% nucleotide identity and 96% - 97% amino acid identity with CARV. Isolates from northeastern Peru and southeastern Peru segmented into distinct lineages within clade I. One lineage was comprised of 35 isolates from northeastern Peru (Iquitos and Yurimaguas), and the second lineage was comprised of one isolate from northeastern Peru and 13 isolates from southeastern Peru (Puerto Maldonado). Clade II comprised ORIV, MURV, APEU, and MTBV strains and 17 clinical isolates, all from northeastern Peru. Isolates shared 83% - 84% nucleotide identity and 97% amino acid identity with MTBV.

**Table 3:** Nucleotide and amino acid sequence identity values for a partial sequence of the S segment from some isolates and reference group C orthobunyaviruses.

	CLADE I												CLADE II							
	Lineage 1						Lineage 2													
	FSL 4095	IQU 1719	FPI 02607	IQE 8988	IQU 2233	ITQV PE 00036	FMD 1692	FMD 1043	FMD 2084	MFI 0919	CARV BeAn 3994	MADV BT 4075	ORIV BeAn 17	MURV BeAn 974	APEUV BeAn 848	MARV BeAn 15	NFI 0798	FLU 3627	IQD 8658	IQT 9891
FSL4095		100.0	100.0	100.0	100.0	98.5	98.5	95.7	95.7	95.0	97.1	95.7	74.4	74.4	75.1	75.8	75.1	75.1	75.1	75.1
IQU1719	99.7		100.0	100.0	100.0	98.5	98.5	95.7	95.7	95.0	97.1	95.7	74.4	74.4	75.1	75.8	75.1	75.1	75.1	75.1
FPI02607	99.0	99.2		100.0	100.0	98.5	98.5	95.7	95.7	95.0	97.1	95.7	74.4	74.4	75.1	75.8	75.1	75.1	75.1	75.1
IQE8988	99.5	99.7	99.5		100.0	98.5	98.5	95.7	95.7	95.0	97.1	95.7	74.4	74.4	75.1	75.8	75.1	75.1	75.1	75.1
IQU2233	96.9	97.1	96.9	97.4		98.5	98.5	95.7	95.7	95.0	97.1	95.7	74.4	74.4	75.1	75.8	75.1	75.1	75.1	75.1
**ITQV PE 00036**	91.0	90.8	90.0	90.5	90.8		98.5	95.7	95.7	95.0	98.5	95.7	75.1	75.1	75.8	76.5	75.8	75.8	75.8	75.8
FMD1692	91.7	91.5	91.2	91.2	90.3	90.0		97.1	97.1	96.4	97.1	95.7	75.1	75.1	75.8	76.5	75.8	75.8	75.8	75.8
FMD1043	91.0	90.8	90.5	90.5	90.8	90.8	93.3		100.0	99.2	97.1	94.3	75.1	75.1	75.8	76.5	75.8	75.8	75.8	75.8
FMD2084	91.0	90.8	90.5	90.5	90.8	90.8	93.3	100.0		99.2	97.1	94.3	75.1	75.1	75.8	76.5	75.8	75.8	75.8	75.8
MFI0919	90.5	90.3	90.0	90.0	90.8	90.3	92.4	98.1	98.1		96.4	93.6	75.1	75.1	75.8	76.5	75.8	75.8	75.8	75.8
**CARV BeAn 3994**	88.4	88.2	87.5	87.9	87.2	86.0	87.7	86.0	86.0	86.7		95.7	75.1	75.1	75.8	76.5	75.8	75.8	75.8	75.8
**MADV BT 4075**	87.2	87.0	86.7	86.7	86.3	83.9	85.8	84.4	84.4	84.6	85.8		73.7	73.7	73.7	75.1	74.4	74.4	74.4	74.4
**ORIV BeAn 17**	73.3	73.5	73.8	73.8	73.5	72.8	73.8	75.0	75.0	74.5	72.1	70.7		100.0	95.0	96.4	96.4	96.4	96.4	96.4
**MURV BeAn 974**	73.1	73.3	73.5	73.5	73.3	72.4	73.3	75.2	75.2	74.2	70.0	69.5	97.1		95.0	96.4	96.4	96.4	96.4	96.4
**APEUV BeAn 848**	70.2	70.0	69.8	69.8	70.0	71.2	71.6	71.6	71.6	71.9	73.5	69.8	82.7	81.8		98.5	95.7	95.7	95.7	95.7
**MARV BeAn 15**	69.1	68.8	69.1	69.1	69.1	71.6	70.9	70.2	70.2	70.9	69.5	70.7	82.5	81.3	86.0		97.1	97.1	97.1	97.1
NFI0798	73.3	73.1	73.3	73.3	72.8	72.8	73.8	74.0	74.0	73.8	72.6	70.0	87.2	86.5	85.1	84.4		100.0	100.0	100.0
FLU3627	73.8	73.5	73.8	73.3	73.1	72.8	74.2	74.0	74.0	72.8	71.4	70.9	86.0	86.7	84.1	83.0	95.5		100.0	100.0
IQD8658	72.8	73.1	73.3	72.8	73.5	72.4	73.8	73.5	73.5	73.3	71.4	71.9	86.5	86.7	84.1	84.4	95.5	98.1		100.0
IQT9891	73.3	73.1	73.3	72.8	72.6	72.4	74.7	74.0	74.0	73.3	71.9	71.4	86.0	86.3	84.6	83.9	95.9	98.5	99.0	

Values in the bottom left half of the data field represent percent nucleotide sequence identity, and values in the top right half represent percent amino acid sequence identity. Reference strains labeled in bold. The percent identity was calculated using 420 nucleotides and 141 amino acids of the partial N and NSs protein.

### M segment sequence analysis

**Fig 3:**
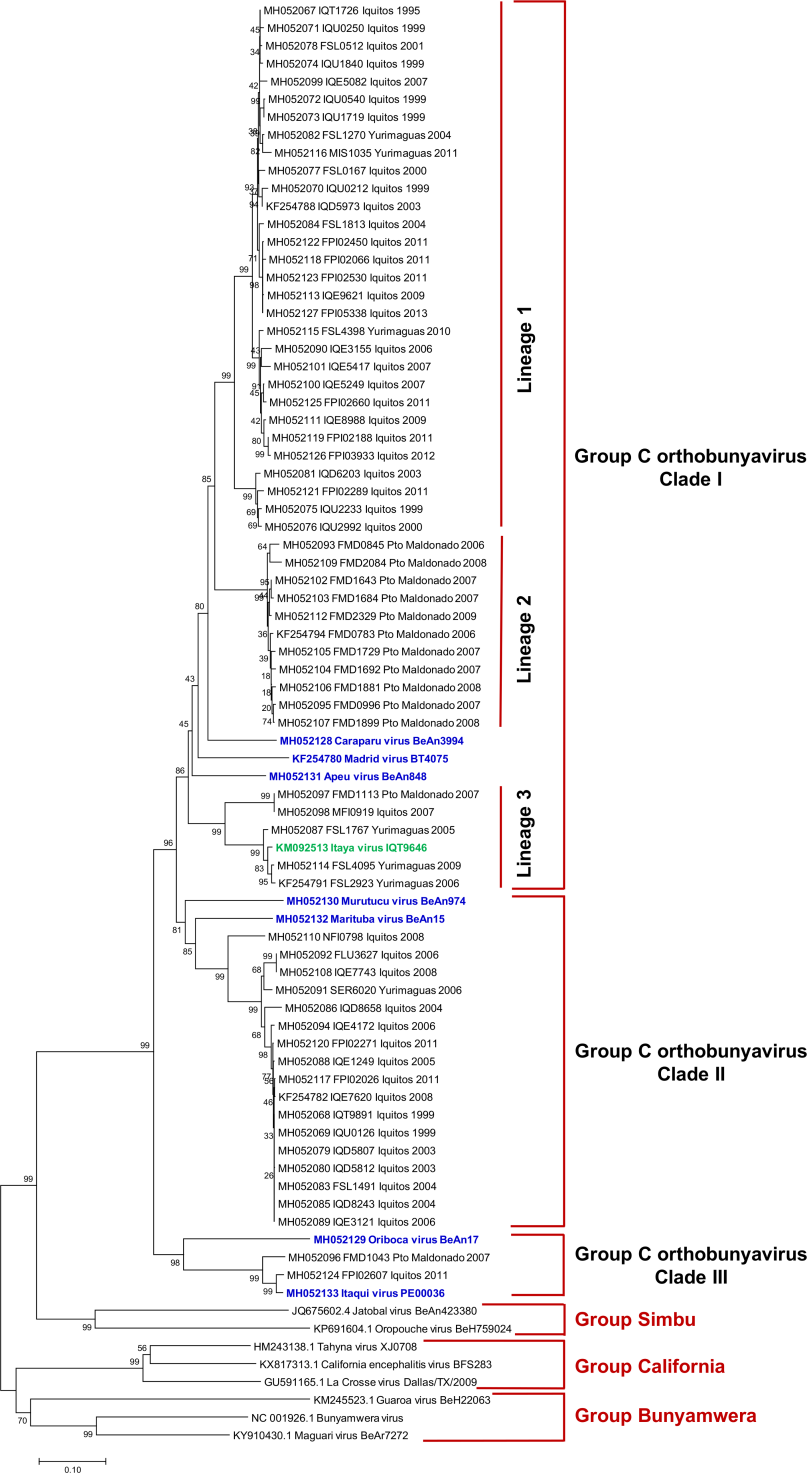
Phylogenetic analysis of group C viruses based on partial nucleotide sequences of the M segment. The phylogenetic tree was constructed using the neighbor-joining method. Bootstrap values were obtained based on 1000 replicates using the partial coding sequence of the polyprotein. The evolutionary distances were computed using the Kimura 2-parameter method. Members of the Simbu, California and Bunyamwera serogroups were used as outgroups to root the tree.

M segment sequences segregated into three clades (Fig 3). Clade I comprised CARV, MADV, APEUV and 47 clinical isolates (including one previously described isolate [17]). Clade I further separated into geographically stratified groupings related to CARV with 79% - 84% nucleotide identity and 90% - 99% amino acid identity (Table 4). Clade I clinical isolates separated into three lineages. Lineage 1 included a cluster of 30 isolates from northeastern Peru. Lineage 2 included a cluster of 11 isolates from southeastern Peru. Lineage 3 included a cluster of 6 isolates from both (northeastern-southeastern Peru). Clade II included MURV, MTBV, and 17 clinical isolates from northeastern Peru, clinical isolates shared 81% - 82% nucleotide identity and 95%– 96% amino acid identity with MTBV. Clade III included ORIV, ITQV, and two clinical isolates (one each from northeastern and southeastern Peru). Clade III clinical isolates were closely related to ITQV (99% - 100% aa identity) and divergent from ORIV (87% aa identity).

**Table 4:** Nucleotide and amino acid sequence identity values for partial sequence of the M segment from some isolates and reference group C orthobunyaviruses.

	CLADE I										CLADE II						CLADE III			
	Lineage 1			Lineage 2					Lineage 3											
	IQU 1719	IQE 8988	IQU 2233	FMD 2084	FMD 1692	CARV BeAn 3994	MADV BT 4075	APEUV BeAn 848	MFI 0919	FSL 4095	MURV BeAn 974	MTBV BeAn 15	NFI 0798	FLU 3627	IQD 8658	IQT 9891	ORIV BeAn 17	FMD 1043	ITQV PE 00036	FPI 02607
IQU1719		100.0	100.0	99.5	100.0	98.7	89.7	94.6	91.8	91.0	85.7	86.5	86.5	86.5	86.1	86.5	73.4	73.4	73.8	73.8
IQE8988	96.6		100.0	99.5	100.0	98.7	89.7	94.6	91.8	91.0	85.7	86.5	86.5	86.5	86.1	86.5	73.4	73.4	73.8	73.8
IQU2233	92.5	91.5		99.5	100.0	98.7	89.7	94.6	91.8	91.0	85.7	86.5	86.5	86.5	86.1	86.5	73.4	73.4	73.8	73.8
FMD2084	84.6	84.5	86.7		99.5	98.3	89.3	94.2	92.2	91.4	85.7	86.5	86.5	86.5	86.1	86.5	73.4	73.4	73.8	73.8
FMD1692	85.3	85.2	88.0	97.1		98.7	89.7	94.6	91.8	91.0	85.7	86.5	86.5	86.5	86.1	86.5	73.4	73.4	73.8	73.8
**CARV BeAn 3994**	83.6	83.3	84.0	82.1	82.7		88.9	93.8	91.0	90.2	84.8	85.3	85.7	85.7	85.3	85.7	73.8	73.4	73.8	73.8
**MADV BT 4075**	79.9	80.2	78.9	78.3	77.7	78.0		88.9	90.6	90.2	84.4	86.1	86.5	86.5	85.7	85.7	73.4	73.0	73.4	73.4
**APEUV BeAn 848**	81.4	81.1	80.2	80.7	80.7	81.1	77.5		91.8	91.0	86.1	86.9	87.3	87.3	87.7	87.3	73.8	75.1	74.6	74.6
MFI0919	79.8	79.1	79.5	77.2	77.7	78.8	76.9	78.4		97.5	86.1	86.1	86.5	86.5	86.5	86.1	73.8	75.9	75.5	75.5
FSL4095	79.5	79.2	80.0	78.7	77.9	79.1	79.2	80.2	87.3		86.1	86.5	87.7	87.7	87.7	87.3	73.0	75.1	74.6	74.6
**MURV BeAn 974**	76.0	75.6	75.3	76.5	75.4	76.8	75.6	77.3	75.0	77.2		91.0	90.6	90.6	91.0	90.2	73.4	73.8	73.4	73.4
**MTBV BeAn 15**	76.6	76.4	76.8	77.2	76.9	77.1	75.4	77.1	77.7	77.3	77.1		95.5	95.5	95.1	95.1	72.2	73.0	73.4	73.4
NFI0798	78.7	77.9	77.2	75.7	76.0	75.6	76.0	77.1	79.1	77.7	77.3	80.6		100.0	99.1	99.1	72.2	73.0	73.4	73.4
FLU3627	76.6	76.8	76.5	75.0	74.7	76.5	76.5	77.6	76.9	77.7	77.9	81.7	88.4		99.1	99.1	72.2	73.0	73.4	73.4
IQD8658	76.2	76.5	76.1	74.9	74.1	75.7	75.3	78.3	76.0	76.8	77.3	80.8	88.6	94.7		99.1	73.0	73.0	72.6	72.6
IQT9891	77.1	77.1	76.9	74.7	74.5	76.6	75.7	78.3	76.4	77.3	78.1	81.1	89.0	96.4	96.6		72.2	72.2	72.6	72.6
**ORIV BeAn 17**	68.5	68.0	68.5	68.6	68.0	68.4	70.8	71.2	69.5	69.1	69.7	71.8	69.7	71.1	70.4	70.5		86.9	86.5	86.5
FMD1043	71.6	71.4	71.8	71.0	71.4	68.9	68.5	70.7	69.2	70.5	69.3	69.5	72.2	70.8	70.4	70.7	73.3		99.1	99.1
**ITQV PE 00036**	71.6	71.5	71.9	70.0	71.2	69.3	69.2	70.5	69.5	69.2	70.3	69.1	71.2	72.3	71.4	71.9	72.4	93.9		100.0
FPI02607	71.5	71.4	71.9	69.6	70.8	69.1	68.8	71.1	69.5	69.3	69.6	69.6	71.4	72.3	71.9	72.2	72.7	94.0	97.9	

Values in the bottom left half of the data field represent the percent nucleotide sequence identity, and values in the top right half represent the percent amino acid sequence identity. Reference strains labeled in bold. The percent identity was calculated using 738 nucleotides and 245 amino acids of the partial polyprotein.

### L segment sequence analysis

**Fig 4:**
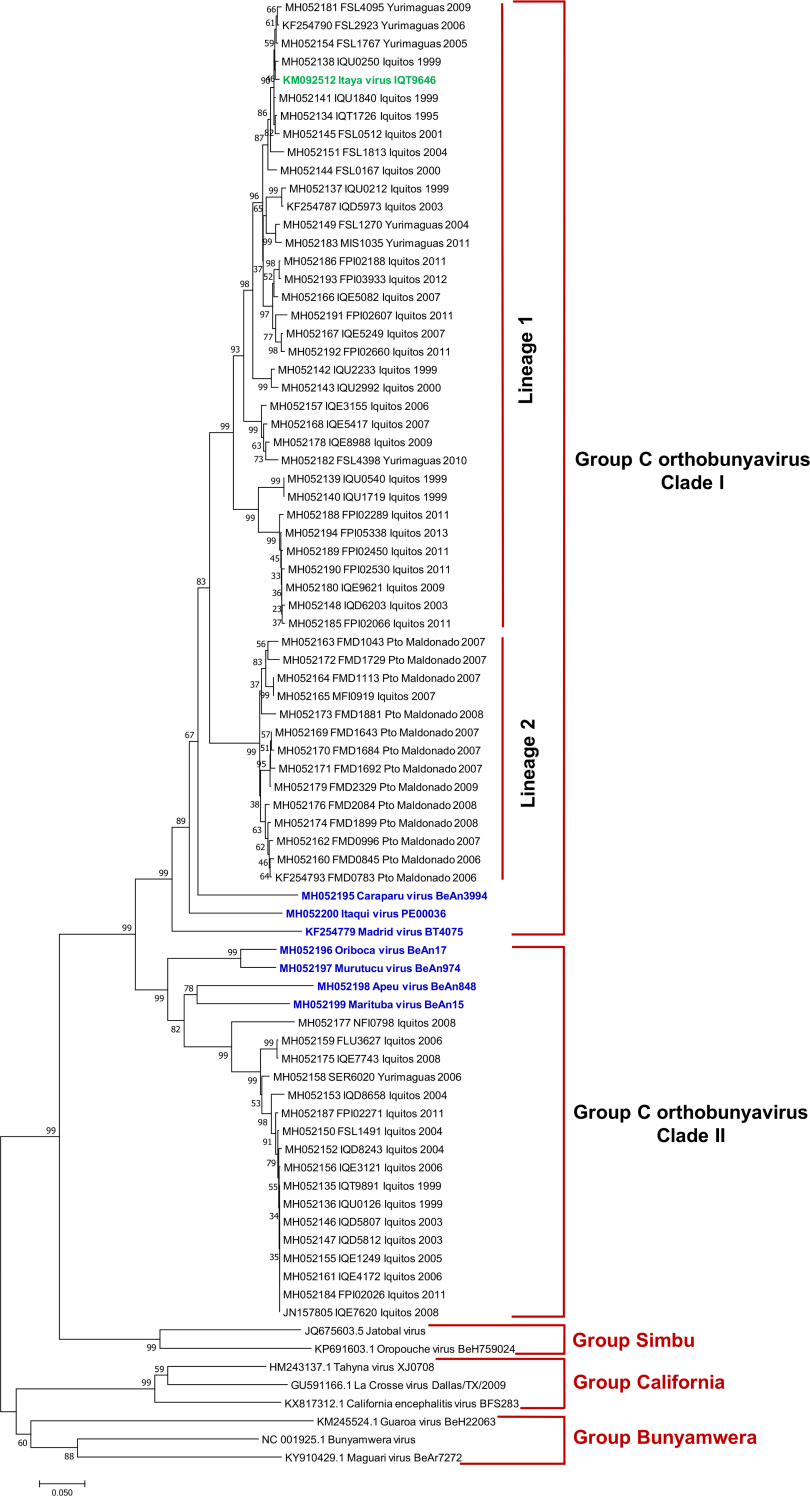
Phylogenetic analysis of group C viruses based on partial nucleotide sequences of the L segment. The phylogenetic tree was constructed using the neighbor-joining method. Bootstrap values were obtained based on 1000 replicates on the bases of partial coding sequence of RdRp. The evolutionary distances were computed using the Kimura 2-parameter method. Members of the Simbu, California and Bunyamwera serogroups were used as outgroups to root the tree.

Clustering of isolates based on L segment analysis mirrored that of the S segment. Based on partial L segment analysis, L segment sequences separated into two clades (Fig 4), with 71% - 76% pairwise nucleotide identity and 78% - 82% pairwise amino acid identity between clade I and clade II isolates (Table 5). As with the S segment, clade I included CARV, MADV, ITQV, and 49 clinical isolates (including one previously described isolate [17]) further segregated into geographically stratified lineages. One lineage included 14 isolates, 13 of which were from southeastern Peru; the other lineage included 35 isolates, all from northeastern Peru. Clade I clinical isolates shared 83% - 84% nucleotide identity and 97% - 98% amino acid identity with CARV. Clade II included ORIV, MURV, MTBV, and APEUV and 17 clinical isolates from northeastern Peru; Clinical isolates shared 80% - 81% nucleotide identity and 95% - 96% amino acid identity with MTBV.

**Table 5:** Nucleotide and amino acid sequence identity values for a partial sequence of the L segment of some isolations and referential group C orthobunyavirus.

	CLADE I												CLADE II							
	Lineage 1					Lineage 2														
	FPI 02607	FSL 4095	IQU 2233	IQE 8988	IQU 1719	FMD 2084	FMD 1692	FMD 1043	MFI 0919	CARV BeAn 3994	ITQV PE 00036	MADV BT 4075	ORIV BeAn 17	MURV BeAn 974	MTBV BeAn 15	APEUV BeAn 848	NFI 0798	FLU 3627	IQD 8658	IQT 9891
FPI02607		99.5	99.5	99.5	99.5	98.7	98.2	98.7	98.7	97.4	97.8	94.8	82.3	82.7	81.8	80.1	81.4	80.6	81.0	81.0
FSL4095	95.4		99.1	99.1	99.1	98.7	98.2	98.7	98.7	97.4	97.8	94.8	82.3	82.7	81.8	80.1	81.4	80.6	81.0	81.0
IQU2233	94.2	94.8		100.0	99.1	98.2	97.8	98.2	98.2	96.9	97.4	95.2	81.8	82.3	81.4	79.7	81.0	80.1	80.6	80.6
IQE8988	93.2	93.8	94.4		99.1	98.2	97.8	98.2	98.2	96.9	97.4	95.2	81.8	82.3	81.4	79.7	81.0	80.1	80.6	80.6
IQU1719	89.2	90.4	90.7	90.8		98.2	97.8	98.2	98.2	97.8	97.4	94.8	82.3	82.7	81.8	80.1	81.4	80.6	81.0	81.0
FMD2084	87.7	87.1	86.7	88.8	85.8		99.5	100.0	100.0	96.9	98.2	95.2	82.3	82.7	81.8	80.6	81.4	80.6	81.0	81.0
FMD1692	87.1	86.5	86.4	88.5	84.8	97.7		99.5	99.5	96.9	98.2	95.6	82.3	82.7	81.8	80.6	81.4	80.6	81.0	81.0
FMD1043	87.1	86.2	85.8	88.0	84.5	96.2	96.4		100.0	96.9	98.2	95.2	82.3	82.7	81.8	80.6	81.4	80.6	81.0	81.0
MFI0919	87.4	86.5	86.1	89.1	85.1	97.1	97.0	97.7		96.9	98.2	95.2	82.3	82.7	81.8	80.6	81.4	80.6	81.0	81.0
**CARV BeAn 3994**	83.0	82.8	82.8	84.1	83.8	83.7	83.1	82.8	83.5		96.9	94.3	81.8	82.3	81.4	80.1	81.0	80.1	80.6	80.6
**ITQV PE 00036**	82.8	82.2	83.4	83.0	82.0	83.1	82.8	83.1	83.1	80.4		94.8	81.8	82.3	81.4	80.1	81.0	80.1	80.6	80.6
**MADV BT 4075**	77.5	77.2	78.7	79.7	79.0	78.4	78.8	79.8	79.4	78.2	78.7		80.1	79.7	79.3	78.8	79.7	79.3	78.8	79.3
**ORIV BeAn 17**	74.5	74.4	74.7	74.8	74.2	75.7	75.5	75.8	75.0	72.1	72.7	73.0		99.5	93.1	91.3	95.2	94.3	94.3	94.8
**MURV BeAn 974**	74.2	74.4	74.7	74.5	74.7	75.2	74.5	75.0	74.0	73.0	74.7	72.2	92.4		93.5	91.8	95.6	94.8	94.8	95.2
**MTBV BeAn 15**	71.8	72.0	73.2	73.7	73.7	73.8	73.7	73.5	74.2	71.5	72.5	71.4	76.4	76.8		94.8	96.1	95.2	94.8	95.6
**APEUV BeAn 848**	72.8	71.7	70.8	71.8	70.4	72.2	71.2	72.5	72.8	71.1	72.0	71.8	75.2	76.4	80.5		94.3	93.5	93.1	93.9
NFI0798	72.8	72.2	73.4	73.5	73.0	73.0	72.1	73.0	72.2	71.8	73.0	72.0	79.4	80.1	80.8	79.0		99.1	98.7	99.5
FLU3627	74.4	74.5	74.1	74.4	74.1	72.8	72.8	74.0	73.2	71.8	72.7	71.7	79.4	79.7	81.1	78.2	89.5		98.7	99.5
IQD8658	73.7	74.4	74.0	73.4	74.0	73.1	73.4	73.7	73.0	71.1	72.1	70.7	78.4	79.0	79.8	78.1	88.1	96.0		99.1
IQT9891	73.8	74.2	73.8	73.5	74.7	73.8	73.7	74.2	73.0	71.8	72.0	71.7	78.8	79.4	80.1	78.5	89.2	96.2	98.0	

Values in the bottom left half of the data field represent the percent nucleotide sequence identity, and values in the top right half represent the percent amino acid sequence identity. Reference strains labeled in bold. The percent identity was calculated using 700 nucleotides and 232 amino acids of the partial RdRp.

### Antigenic characterization

Clinical isolates fell into four antigenically distinct groups (Table 6) which tracked closely with M segment sequence phylogeny. CARV HMAF had the highest titers against 41 clinical isolates (titers ranging from 1/40 to 1/640.) All 41 had M segment 98% - 99% amino acid identity with CARV. In some cases, these isolates also tended to show some limited cross-reactivity with APEUV, MTBV, or MURV HMAF (typically titers of 1/20 or 1/40). Seventeen isolates reacted robustly against MTBV and MURV HMAF (titers ranging from 1/160 to 1/1280; median titer 1/320), with no detected cross-reactivity with other reference strains. Five strains displayed only low titers against MTBV HMAF (1/20 or 1/40) and no detectable titers against other strains. Two isolates displayed highest titers against ITQV HMAF. No isolates displayed high titers with ORIV HMAF.

**Table 6:** Microneutralization test of group C orthobunyavirus (GRCV) using hyperimmune mouse ascitic fluids (HMAFs) prepared with prototype strains.

Year	Code	APEUV HMAF	CARV HMAF	ITQV (PE00036) HMAF	MTBV HMAF	MURV HMAF	ORIV HMAF	Complex
1995	IQT1726	NEG	**1/80**	NEG	NEG	NEG	NEG	**CARV**
1999	IQU0250	NEG	**1/40**	NEG	NEG	NEG	NEG	**CARV**
1999	IQU0540	NEG	**1/320**	NEG	NEG	NEG	NEG	**CARV**
1999	IQU1719	NEG	**1/80**	NEG	NEG	NEG	NEG	**CARV**
1999	IQU1840	NEG	**1/40**	NEG	NEG	NEG	NEG	**CARV**
1999	IQU2233	NEG	**1/80**	NEG	NEG	NEG	NEG	**CARV**
2000	IQU2992	NEG	**1/40**	NEG	NEG	NEG	NEG	**CARV**
2006	FMD0783	NEG	**1/80**	NEG	NEG	NEG	NEG	**CARV**
2006	IQE3155	NEG	**1/80**	NEG	NEG	NEG	NEG	**CARV**
2007	FMD1692	NEG	**1/80**	NEG	NEG	NEG	NEG	**CARV**
2007	FMD1729	NEG	**1/40**	NEG	NEG	NEG	NEG	**CARV**
2007	IQE5249	NEG	**1/80**	NEG	NEG	NEG	NEG	**CARV**
2007	IQE5417	NEG	**1/80**	NEG	NEG	NEG	NEG	**CARV**
2008	FMD2084	NEG	**1/40**	NEG	NEG	NEG	NEG	**CARV**
2009	FMD2329	NEG	**1/80**	NEG	NEG	NEG	NEG	**CARV**
2009	IQE9621	NEG	**1/160**	NEG	NEG	NEG	NEG	**CARV**
2010	FSL4398	NEG	**1/80**	NEG	NEG	NEG	NEG	**CARV**
2011	FPI02066	NEG	**1/80**	NEG	NEG	NEG	NEG	**CARV**
2011	FPI02188	NEG	**1/80**	NEG	NEG	NEG	NEG	**CARV**
2011	FPI02660	NEG	**1/80**	NEG	NEG	NEG	NEG	**CARV**
1999	IQU0212	**1/20**	**1/80**	NEG	NEG	NEG	NEG	**CARV-APEUV**
2003	IQD5973	**1/20**	**1/80**	NEG	NEG	NEG	NEG	**CARV-APEUV**
2004	FSL1813	**1/20**	**1/160**	NEG	NEG	NEG	NEG	**CARV-APEUV**
2008	FMD1899	**1/20**	**1/80**	NEG	NEG	NEG	NEG	**CARV-APEUV**
2012	FPI3933	**1/320**	**1/640**	NEG	NEG	NEG	NEG	**CARV-APEUV**
2013	FPI5338	**1/320**	**1/640**	NEG	NEG	NEG	NEG	**CARV-APEUV**
2000	FSL0167	**1/20**	**1/320**	NEG	**1/20**	NEG	NEG	**CARV-APEUV-MTBV**
2007	FMD1643	**1/20**	**1/160**	NEG	**1/20**	NEG	NEG	**CARV-APEUV-MTBV**
2007	FMD1684	**1/40**	**1/160**	NEG	**1/20**	NEG	NEG	**CARV-APEUV-MTBV**
2007	IQE5082	**1/40**	**1/160**	NEG	**1/20**	NEG	NEG	**CARV-APEUV-MTBV**
2008	FMD1881	**1/40**	**1/160**	NEG	**1/20**	NEG	NEG	**CARV-APEUV-MTBV**
2009	IQE8988	**1/20**	**1/320**	NEG	**1/20**	NEG	NEG	**CARV-APEUV-MTBV**
2011	FPI02530	**1/40**	**1/160**	NEG	**1/20**	NEG	NEG	**CARV-APEUV-MTBV**
2007	FMD0996	**1/40**	**1/320**	NEG	**1/40**	**1/20**	NEG	**CARV-APEUV-MTBV-MURV**
2011	FPI02450	**1/80**	**1/320**	NEG	**1/40**	**1/20**	NEG	**CARV-APEUV-MTBV-MURV**
2011	MIS1035	**1/40**	**1/640**	NEG	**1/40**	**1/40**	NEG	**CARV-APEUV-MTBV-MURV**
2001	FSL0512	NEG	**1/160**	NEG	**1/20**	NEG	NEG	**CARV-MTBV**
2003	IQD6203	NEG	**1/160**	NEG	**1/40**	NEG	NEG	**CARV-MTBV**
2004	FSL1270	NEG	**1/320**	NEG	**1/40**	NEG	NEG	**CARV-MTBV**
2011	FPI02289	NEG	**1/160**	NEG	**1/20**	NEG	NEG	**CARV-MTBV**
2006	FMD0845	NEG	**1/320**	NEG	**1/40**	**1/20**	NEG	**CARV-MTBV-MURV**
2005	FSL1767	NEG	NEG	NEG	**1/20**	NEG	NEG	**MTBV**
2006	FSL2923	NEG	NEG	NEG	**1/40**	NEG	NEG	**MTBV**
2007	FMD1113	NEG	NEG	NEG	**1/40**	NEG	NEG	**MTBV**
2007	MFI0919	NEG	NEG	NEG	**1/20**	NEG	NEG	**MTBV**
2009	FSL4095	NEG	NEG	NEG	**1/20**	NEG	NEG	**MTBV**
1999	IQT9891	NEG	NEG	NEG	**1/160**	**1/160**	NEG	**MTBV-MURV**
1999	IQU0126	NEG	NEG	NEG	**1/160**	**1/160**	NEG	**MTBV-MURV**
2003	IQD5807	NEG	NEG	NEG	**1/640**	**1/320**	NEG	**MTBV-MURV**
2003	IQD5812	NEG	NEG	NEG	**1/640**	**1/320**	NEG	**MTBV-MURV**
2004	FSL1491	NEG	NEG	NEG	**1/1280**	**1/1280**	NEG	**MTBV-MURV**
2004	IQD8243	NEG	NEG	NEG	**1/640**	**1/320**	NEG	**MTBV-MURV**
2004	IQD8658	NEG	NEG	NEG	**1/640**	**1/640**	NEG	**MTBV-MURV**
2005	IQE1249	NEG	NEG	NEG	**1/160**	**1/320**	NEG	**MTBV-MURV**
2006	IQE3121	NEG	NEG	NEG	**1/320**	**1/320**	NEG	**MTBV-MURV**
2006	IQE4172	NEG	NEG	NEG	**1/160**	**1/320**	NEG	**MTBV-MURV**
2006	SER6020	NEG	NEG	NEG	**1/160**	**1/160**	NEG	**MTBV-MURV**
2006	FLU3627	NEG	NEG	NEG	**1/320**	**1/320**	NEG	**MTBV-MURV**
2008	IQE7620	NEG	NEG	NEG	**1/640**	**1/640**	NEG	**MTBV-MURV**
2008	IQE7743	NEG	NEG	NEG	**1/640**	**1/640**	NEG	**MTBV-MURV**
2008	NFI0798	NEG	NEG	NEG	**1/160**	**1/160**	NEG	**MTBV-MURV**
2011	FPI02026	NEG	NEG	NEG	**1/320**	**1/160**	NEG	**MTBV-MURV**
2011	FPI02271	NEG	NEG	NEG	**1/320**	**1/160**	NEG	**MTBV-MURV**
2011	FPI02607	NEG	NEG	**1/640**	NEG	NEG	NEG	**ITQV (PE00036)**
2007	FMD1043	NEG	NEG	**1/320**	NEG	NEG	**1/20**	**ITQV (PE00036)-ORIV**
1955	**APEUV BeAn848**	**1/320**	NEG	NEG	NEG	NEG	NEG	**APEUV**
1956	**CARV BeAn3994**	NEG	**1/80**	NEG	NEG	NEG	NEG	**CARV**
1996	**ITQV (PE00036)**	NEG	NEG	**1/320**	NEG	NEG	NEG	**ITQV (PE00036)**
1954	**MTBV BeAn15**	NEG	**1/20**	**1/20**	**1/2560**	**1/160**	NEG	**MTBV-MURV**
1955	**MURV BeAn974**	NEG	NEG	NEG	**1/320**	**1/320**	NEG	**MTBV-MURV**
1954	**ORIV BeAn17**	NEG	NEG	**1/20**	NEG	NEG	**1/160**	**ORIV**

Indicated values are neutralization titers. With pink background are the group of isolates that were neutralized with highest titer by CAR HMAF and also belongs to linage 1 and 2 from clade I in the M segment sequence phylogeny. With orange background are the group of isolates that were neutralized with very low titer with MTBV HMAF and belong to linage 3 from clade I in the M segment sequence phylogeny. With green background are the group of isolates that were neutralized by MTBV- MURV and belongs to clade II in the M segment sequence phylogeny. With yellow background are the isolates that were neutralized with highest titer by ITQV and belongs to clade III in the M segment sequence phylogeny.

### Categorization of isolates

The genetic characteristics of reference strains CARV BeAn3994 and MADV BT4075 tracked together for S, M, and L in clade I, and MURV BeAn974 and MTBV BeAn15 tracked together for S, M, and L in clade II. ITQV PE00036 tracked with CARV BeAn3994 and MADV BT4075 for S and L as part of clade I, but with ORIV BeAn17 for M, as part of clade III. APEUV BeAn848 and ORIV BeAn17 tracked with MURV BeAn974 and MTBV BeAn15 for S and L segments as part of clade II, but APEUV BeAn848 tracked with CARV BeAn3994 for the M segment as part of the clade I, and ORIV BeAn17 tracked with ITQV PE00036 for M, as part of clade III. These results are based on neighbor-joining method; similar topologies were identified using maximum likelihood and maximum parsimony approaches (S1–S6 Figs).

Based on genetic and antigenic characteristics, clinical isolates could be grouped with prototype strains. Specifically, 41 isolates were most closely related to CARV (amino acid identity > = 96% S, > = 98% M, > = 97% L, and highest neutralization titers with CARV HMAF), 17 isolates were most closely related to MURV-MTBV serocomplex (MTBV amino acid identity > = 97% S, > = 95% M, > = 95% L, and highest neutralization titers with MURV or MTBV HMAF), and 2 with ITQV (amino acid identity > = 96% S, > = 99% M, > = 98% L). No ORIV isolates were detected. Additionally, six reassortants relative to CARV reference strains were identified (90% - 91% M segment amino acid identity). These isolates grouped with Itaya virus, a novel reassortant orthobunyavirus isolated from febrile patients in the cities of Iquitos and Yurimaguas in Peru [16,17]. Itaya virus S and L segments are closely related to the respective segments of CARV; in contrast, the Itaya virus M segment is distinct from currently characterized orthobunyaviruses.

## Discussion

In this study, we identified 41 isolates of CARV, 17 isolates of MURV, 6 isolates of Itaya virus, and 2 isolates of ITQV associated with human febrile illness in the northern and southern Amazon basin of Peru. The ITQV isolates represent the first association of ITQV with human illness in Peru. Overall, while the Peruvian isolates were most closely related genetically to the indicated reference viruses, they still displayed greater than 10% divergence at the nucleotide level in comparison to the reference viruses, consistent with the fact that the reference viruses were collected in neighboring Brazil several decades earlier.

Our data confirm that the M segment gene product is the key determinant of virus neutralization specificity. Similarly, early studies on GRCVs [1,3,4] described cross-reaction based on hemagglutination inhibition assays and neutralization testing between CARV—APEUV, MARV—MURV and ORIV–ITQV and cross reaction based on complement fixation assays between MURV- ORIV, ITQV—CARV and APEUV—MTBV [3,4]. Our phylogenetic results are consistent with the notion that antibodies detected by hemagglutination inhibition assay and neutralization testing are made in response to surface glycoproteins encoded on the M segment, and antibodies detected by complement fixation are made in response to the nucleocapsid protein encoded on the S segment [25].

For all isolates described here, S and L segments segregated together, both grouping into two major clades. For S and L, clade I was divided into two lineages, with lineages most closely aligned with geographic distribution rather than year of isolation. Lineages 1 and 3 of clade I contained only isolates from the northern Amazon region of Peru, while lineage 2 mainly contained isolates from the southern Amazon region of Peru. These data suggest that there has been little flow of orthobunyaviruses within Peru over the past 20 years.

While the majority of isolates grouped consistently across all three segments, there were clear inconsistencies between S and L versus M segment for a subset of viruses, likely representing reassortants. In this virus set, six isolates of Itaya virus were identified, including four new cases in addition to the originally identified isolate [16] and the isolate characterized by Hontz et al [17]. Notably, two reference viruses, APEUV and ORIV, also appear to be reassortants. APEUV grouped with MTBV and related isolates for the S and L segments. However, APEUV grouped with CARV and MADV for the M segment, and all isolates that were neutralized by APEUV HMAF were also neutralized by CARV. ORIV grouped with MURV for the S and L segments but with ITQV for the M segment. There also appeared to be some serological cross-reaction between ORIV and ITQV.

These data demonstrate the diversity of GRCVs that infect and cause febrile illness in humans in the Peruvian Amazon. Our data also suggest that reassortment among GRCVs is a relatively common phenomenon, as described for other bunyaviruses [26]. Further ecological studies are warranted to better understand the arthropod vectors and vertebrate hosts that maintain these viruses in the Peru and potential for broader emergence, particularly in light of the known impact of other orthobunyaviruses in human (e.g., Oropouche virus) and livestock (e.g.,Schmallenburg virus) disease.

## Supporting information

S1 Table Group C orthobunyavirus (GRCV), Peruvian isolates used in the study. (DOCX)

S1 Fig Phylogenetic analysis by Maximum Likelihood method of group C viruses based on partial nucleotide sequences of the S segment. The phylogenetic tree was constructed using the Maximum Likelihood method based on the Kimura 2-parameter model. Bootstrap values were inferred from 500 replicates on the bases of partial coding sequence of N and NSs protein. Members of the Simbu, California and Bunyamwera serogroups were used as outgroups to root the tree.
(TIF)

S2 Fig Maximum Parsimony (MP) analysis of group C viruses based on partial nucleotide sequences of the S segment. The MP tree was constructed using the Maximum Parsimony method. Bootstrap consensus tree inferred from 500 replicates is taken to represent the evolutionary history of the taxa analyzed on the bases of partial coding sequence of N and NSs protein. The MP tree was obtained using the Subtree-Pruning-Regrafting (SPR) algorithm. Members of the Simbu, California and Bunyamwera serogroups were used as outgroups to root the tree.
(TIF)

S3 Fig Phylogenetic analysis by Maximum Likelihood method of group C viruses based on partial nucleotide sequences of the M segment. The phylogenetic tree was constructed using the Maximum Likelihood method based on the Kimura 2-parameter model. Bootstrap values were inferred from 500 replicates on the bases of partial coding sequence of the polyprotein. Members of the Simbu, California and Bunyamwera serogroups were used as outgroups to root the tree.
(TIF)

S4 Fig Maximum Parsimony (MP) analysis of group C viruses based on partial nucleotide sequences of the M segment. The MP tree was constructed using the Maximum Parsimony method. Bootstrap consensus tree inferred from 500 replicates is taken to represent the evolutionary history of the taxa analyzed on the bases of partial coding sequence of the polyprotein. The MP tree was obtained using the Subtree-Pruning-Regrafting (SPR) algorithm. Members of the Simbu, California and Bunyamwera serogroups were used as outgroups to root the tree.
(TIF)

S5 Fig Phylogenetic analysis by Maximum Likelihood method of group C viruses based on partial nucleotide sequences of the L segment. The phylogenetic tree was constructed using the Maximum Likelihood method based on the Kimura 2-parameter model. Bootstrap values were inferred from 500 replicates on the bases of partial coding sequence of RdRp. Members of the Simbu, California and Bunyamwera serogroups were used as outgroups to root the tree.
(TIF)

S6 Fig Maximum Parsimony (MP) analysis of group C viruses based on partial nucleotide sequences of the L segment. The MP tree was constructed using the Maximum Parsimony method. Bootstrap consensus tree inferred from 500 replicates is taken to represent the evolutionary history of the taxa analyzed on the bases of partial coding sequence of RdRp. The MP tree was obtained using the Subtree-Pruning-Regrafting (SPR) algorithm. Members of the Simbu, California and Bunyamwera serogroups were used as outgroups to root the tree.
(TIF)
