# Supplementary material for: Molecular and antigenic characterization of group C orthobunyaviruses isolated in Peru
Source: PLoS One. 2018 Jul 19;13(7):e0200576. doi: 10.1371/journal.pone.0200576 (PMC6053143; doi:10.1371/journal.pone.0200576)
Supplement: S1 Table — (DOCX) [file pone.0200576.s001.docx]

S1 Table. Group C orthobunyavirus (GRCV) Peruvian isolates used in the study

| **Strain** | **Year** | **City *** | **Age** | **Gender** | **GenBank Accession Numbers (Segments S, M and L)** |
| --- | --- | --- | --- | --- | --- |
| IQT1726 | 1995 | Iquitos | 40 | Male | MH052000, MH052067, MH052134 |
| IQT9891 | 1999 | Iquitos | 20 | Male | MH052001, MH052068, MH052135 |
| IQU0126 | 1999 | Iquitos | 11 | Male | MH052002, MH052069, MH052136 |
| IQU0212 | 1999 | Iquitos | 17 | Male | MH052003, MH052070, MH052137 |
| IQU0250 | 1999 | Iquitos | 34 | Female | MH052004, MH052071, MH052138 |
| IQU0540 | 1999 | Iquitos | 18 | Female | MH052005, MH052072, MH052139 |
| IQU1719 | 1999 | Iquitos | 24 | Male | MH052006, MH052073, MH052140 |
| IQU1840 | 1999 | Iquitos | 13 | Female | MH052007, MH052074, MH052141 |
| IQU2233 | 1999 | Iquitos | 36 | Female | MH052008, MH052075, MH052142 |
| IQU2992 | 2000 | Iquitos | 23 | Female | MH052009, MH052076, MH052143 |
| FSL0167 | 2000 | Iquitos | 33 | Male | MH052010, MH052077, MH052144 |
| FSL0512 | 2001 | Iquitos | 39 | Male | MH052011, MH052078, MH052145 |
| IQD5807 | 2003 | Iquitos | 16 | Female | MH052012, MH052079, MH052146 |
| IQD5812 | 2003 | Iquitos | 59 | Male | MH052013, MH052080, MH052147 |
| IQD5973 | 2003 | Iquitos | 17 | Female | KF254789, KF254788, KF254787 |
| IQD6203 | 2003 | Iquitos | 34 | Male | MH052014, MH052081, MH052148 |
| FSL1270 | 2004 | Yurimaguas | 20 | Male | MH052015, MH052082, MH052149 |
| FSL1491 | 2004 | Iquitos | 24 | Male | MH052016, MH052083, MH052150 |
| FSL1813 | 2004 | Iquitos | 24 | Female | MH052017, MH052084, MH052151 |
| IQD8243 | 2004 | Iquitos | 16 | Male | MH052018, MH052085, MH052152 |
| IQD8658 | 2004 | Iquitos | 48 | Female | MH052019, MH052086, MH052153 |
| FSL1767 | 2005 | Yurimaguas | 13 | Male | MH052020, MH052087, MH052154 |
| IQE1249 | 2005 | Iquitos | 31 | Female | MH052021, MH052088, MH052155 |
| IQE3121 | 2006 | Iquitos | 6 | Female | MH052022, MH052089, MH052156 |
| IQE3155 | 2006 | Iquitos | 27 | Male | MH052023, MH052090, MH052157 |
| FMD0783 | 2006 | Puerto Maldonado | 21 | Male | KF254795, KF254794, KF254793 |
| SER6020 | 2006 | Yurimaguas | 12 | Male | MH052024, MH052091, MH052158 |
| FLU3627 | 2006 | Iquitos | 27 | Female | MH052025, MH052092, MH052159 |
| FMD0845 | 2006 | Puerto Maldonado | 38 | Male | MH052026, MH052093, MH052160 |
| IQE4172 | 2006 | Iquitos | 19 | Female | MH052027, MH052094, MH052161 |
| FSL2923 | 2006 | Yurimaguas | 59 | Male | KF254792, KF254791, KF254790 |
| FMD0996 | 2007 | Puerto Maldonado | 21 | Male | MH052028, MH052095, MH052162 |
| FMD1043 | 2007 | Puerto Maldonado | 36 | Male | MH052029, MH052096, MH052163 |
| FMD1113 | 2007 | Puerto Maldonado | 15 | Male | MH052030, MH052097, MH052164 |
| MFI0919 | 2007 | Iquitos | 25 | Male | MH052031, MH052098, MH052165 |
| IQE5082 | 2007 | Iquitos | 20 | Female | MH052032, MH052099, MH052166 |
| IQE5249 | 2007 | Iquitos | 29 | Male | MH052033, MH052100, MH052167 |
| IQE5417 | 2007 | Iquitos | 27 | Male | MH052034, MH052101, MH052168 |
| FMD1643 | 2007 | Puerto Maldonado | 25 | Male | MH052035, MH052102, MH052169 |
| FMD1684 | 2007 | Puerto Maldonado | 21 | Male | MH052036, MH052103, MH052170 |
| FMD1692 | 2007 | Puerto Maldonado | 19 | Male | MH052037, MH052104, MH052171 |
| FMD1729 | 2007 | Puerto Maldonado | 44 | Female | MH052038, MH052105, MH052172 |
| FMD1881 | 2008 | Puerto Maldonado | 28 | Male | MH052039, MH052106, MH052173 |
| FMD1899 | 2008 | Puerto Maldonado | 38 | Male | MH052040, MH052107, MH052174 |
| IQE7620 | 2008 | Iquitos | 14 | Female | KF254783, KF254782, JN157805 |
| IQE7743 | 2008 | Iquitos | 46 | Female | MH052041, MH052108, MH052175 |
| FMD2084 | 2008 | Puerto Maldonado | 35 | Male | MH052042, MH052109, MH052176 |
| NFI0798 | 2008 | Iquitos | 36 | Male | MH052043, MH052110, MH052177 |
| IQE8988 | 2009 | Iquitos | 20 | Female | MH052044, MH052111, MH052178 |
| FMD2329 | 2009 | Puerto Maldonado | 22 | Male | MH052045, MH052112, MH052179 |
| IQE9621 | 2009 | Iquitos | 63 | Female | MH052046, MH052113, MH052180 |
| FSL4095 | 2009 | Yurimaguas | 50 | Male | MH052047, MH052114, MH052181 |
| FSL4398 | 2010 | Yurimaguas | 47 | Female | MH052048, MH052115, MH052182 |
| MIS1035 | 2011 | Yurimaguas | 9 | Male | MH052049, MH052116, MH052183 |
| FPI02026 | 2011 | Iquitos | 36 | Female | MH052050, MH052117, MH052184 |
| FPI02066 | 2011 | Iquitos | 29 | Male | MH052051, MH052118, MH052185 |
| FPI02188 | 2011 | Iquitos | 32 | Male | MH052052, MH052119, MH052186 |
| FPI02271 | 2011 | Iquitos | 27 | Male | MH052053, MH052120, MH052187 |
| FPI02289 | 2011 | Iquitos | 19 | Male | MH052054, MH052121, MH052188 |
| FPI02450 | 2011 | Iquitos | 19 | Female | MH052055, MH052122, MH052189 |
| FPI02530 | 2011 | Iquitos | 33 | Female | MH052056, MH052123, MH052190 |
| FPI02607 | 2011 | Iquitos | 52 | Female | MH052057, MH052124, MH052191 |
| FPI02660 | 2011 | Iquitos | 18 | Male | MH052058, MH052125, MH052192 |
| FPI03933 | 2012 | Iquitos | 42 | Female | MH052059, MH052126, MH052193 |
| FPI05338 | 2013 | Iquitos | 17 | Male | MH052060, MH052127, MH052194 |

*Isolations were made in the city or nearby
