# Supplementary figures and images for: Molecular and antigenic characterization of group C orthobunyaviruses isolated in Peru
Source: PLoS One. 2018 Jul 19;13(7):e0200576. doi: 10.1371/journal.pone.0200576 (PMC6053143; doi:10.1371/journal.pone.0200576)

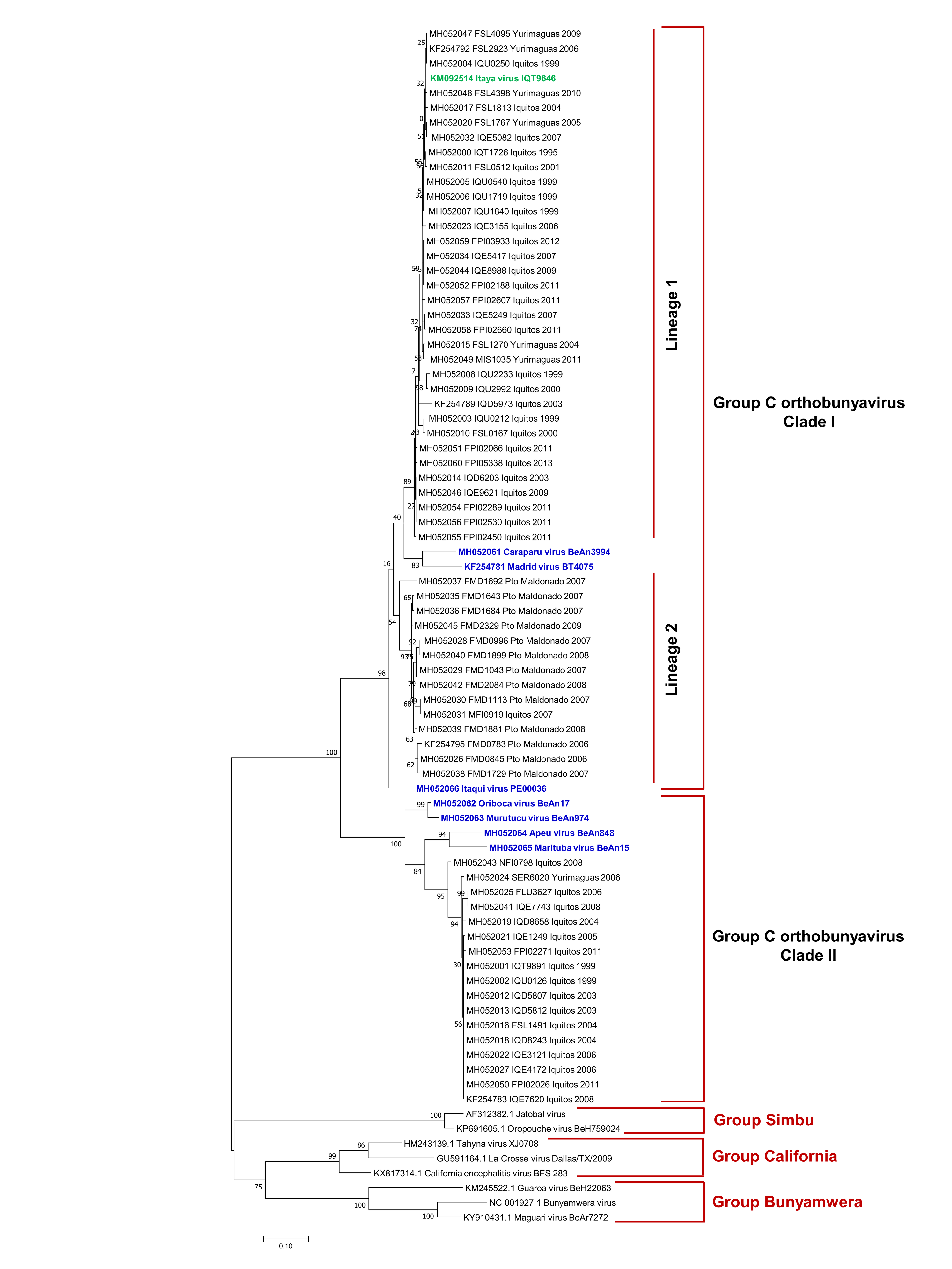

Supplement: S1 Fig — The phylogenetic tree was constructed using the Maximum Likelihood method based on the Kimura 2-parameter model. Bootstrap values were inferred from 500 replicates on the bases of partial coding sequence of N and NSs protein. Members of the Simbu, California and Bunyamwera serogroups were used as outgroups to root the tree. (TIF) [file pone.0200576.s002.tif]

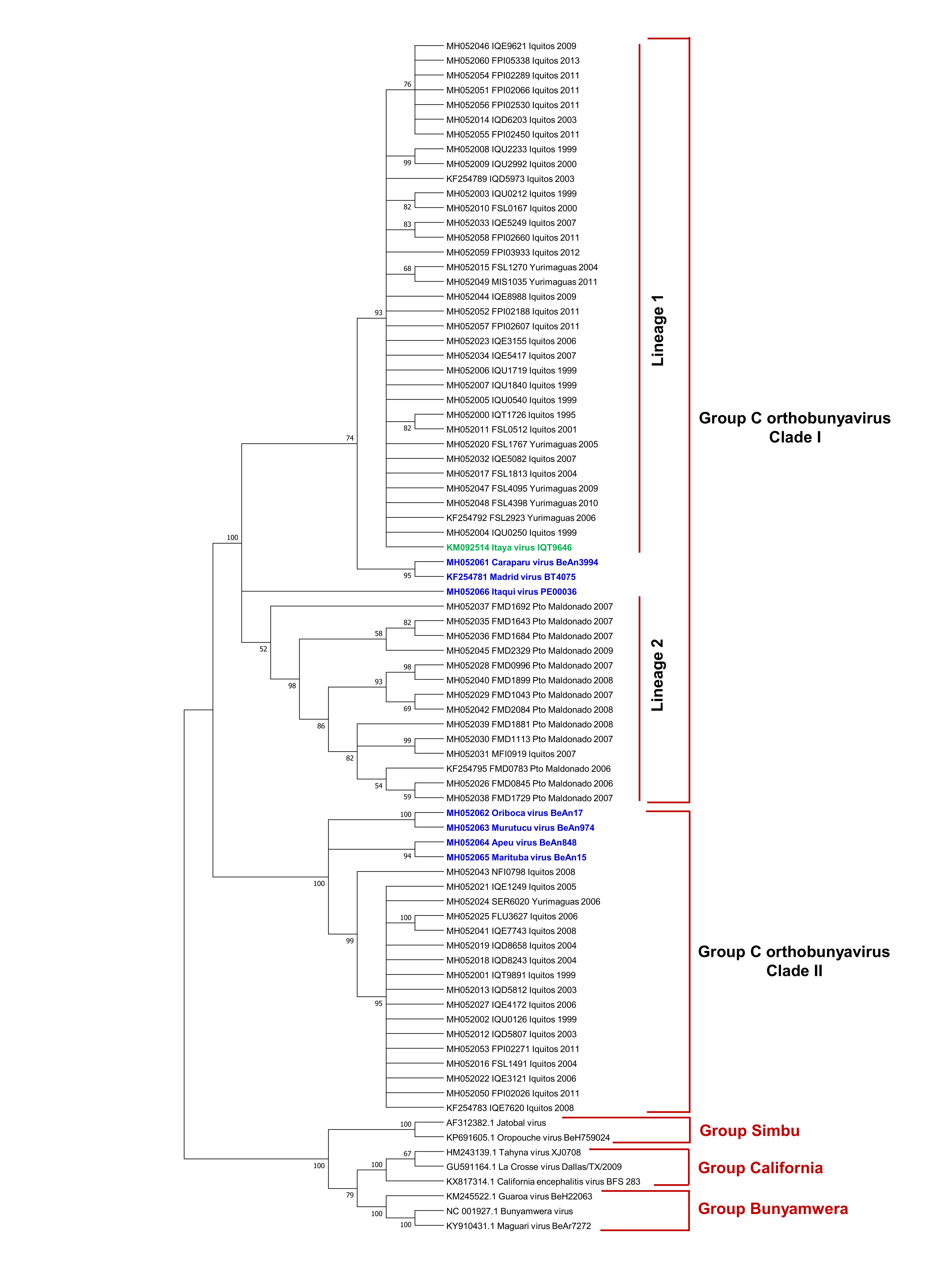

Supplement: S2 Fig — The MP tree was constructed using the Maximum Parsimony method. Bootstrap consensus tree inferred from 500 replicates is taken to represent the evolutionary history of the taxa analyzed on the bases of partial coding sequence of N and NSs protein. The MP tree was obtained using the Subtree-Pruning-Regrafting (SPR) algorithm. Members of the Simbu, California and Bunyamwera serogroups were used as outgroups to root the tree. (TIF) [file pone.0200576.s003.tif]

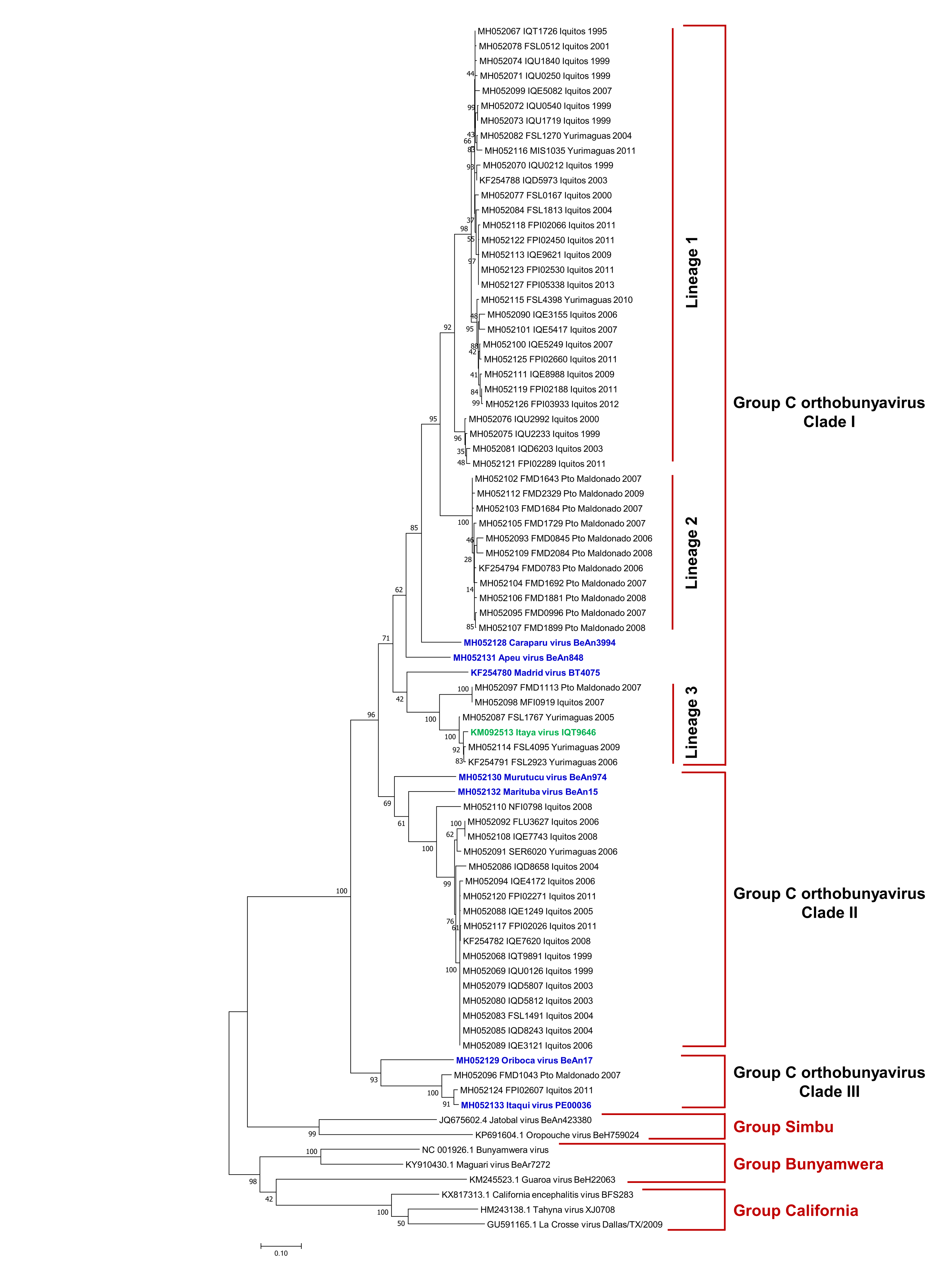

Supplement: S3 Fig — The phylogenetic tree was constructed using the Maximum Likelihood method based on the Kimura 2-parameter model. Bootstrap values were inferred from 500 replicates on the bases of partial coding sequence of the polyprotein. Members of the Simbu, California and Bunyamwera serogroups were used as outgroups to root the tree. (TIF) [file pone.0200576.s004.tif]

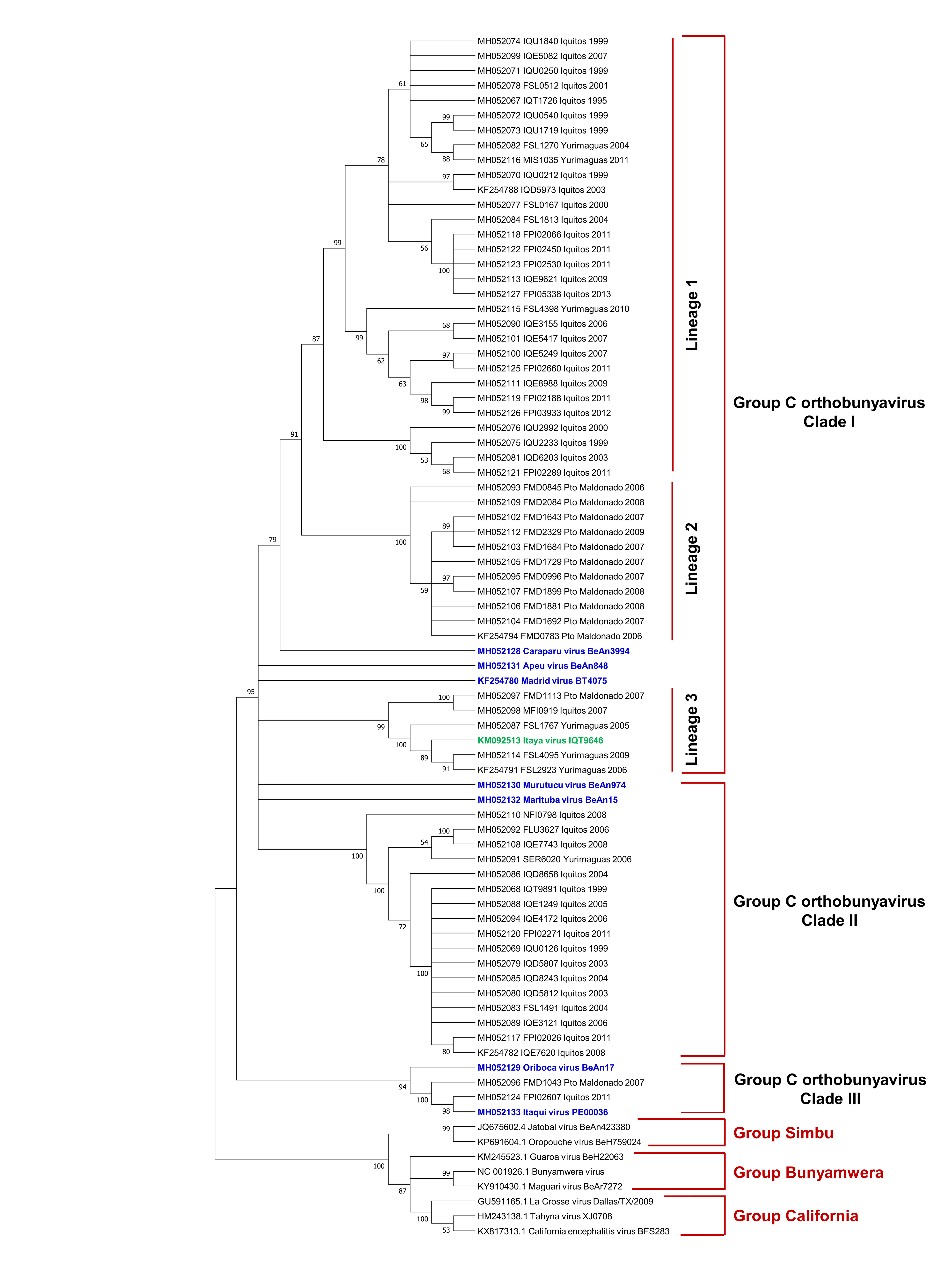

Supplement: S4 Fig — The MP tree was constructed using the Maximum Parsimony method. Bootstrap consensus tree inferred from 500 replicates is taken to represent the evolutionary history of the taxa analyzed on the bases of partial coding sequence of the polyprotein. The MP tree was obtained using the Subtree-Pruning-Regrafting (SPR) algorithm. Members of the Simbu, California and Bunyamwera serogroups were used as outgroups to root the tree. (TIF) [file pone.0200576.s005.tif]

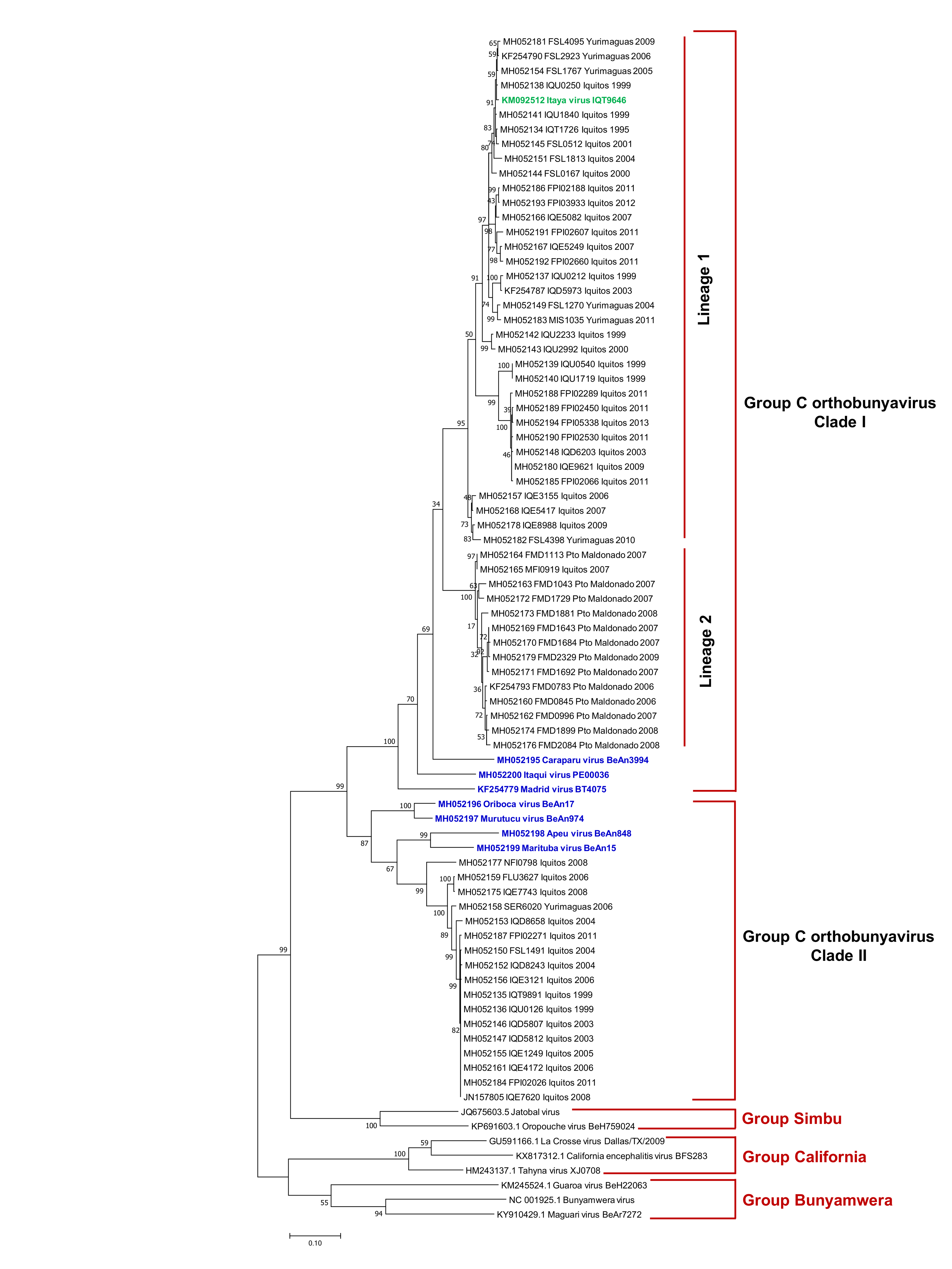

Supplement: S5 Fig — The phylogenetic tree was constructed using the Maximum Likelihood method based on the Kimura 2-parameter model. Bootstrap values were inferred from 500 replicates on the bases of partial coding sequence of RdRp. Members of the Simbu, California and Bunyamwera serogroups were used as outgroups to root the tree. (TIF) [file pone.0200576.s006.tif]

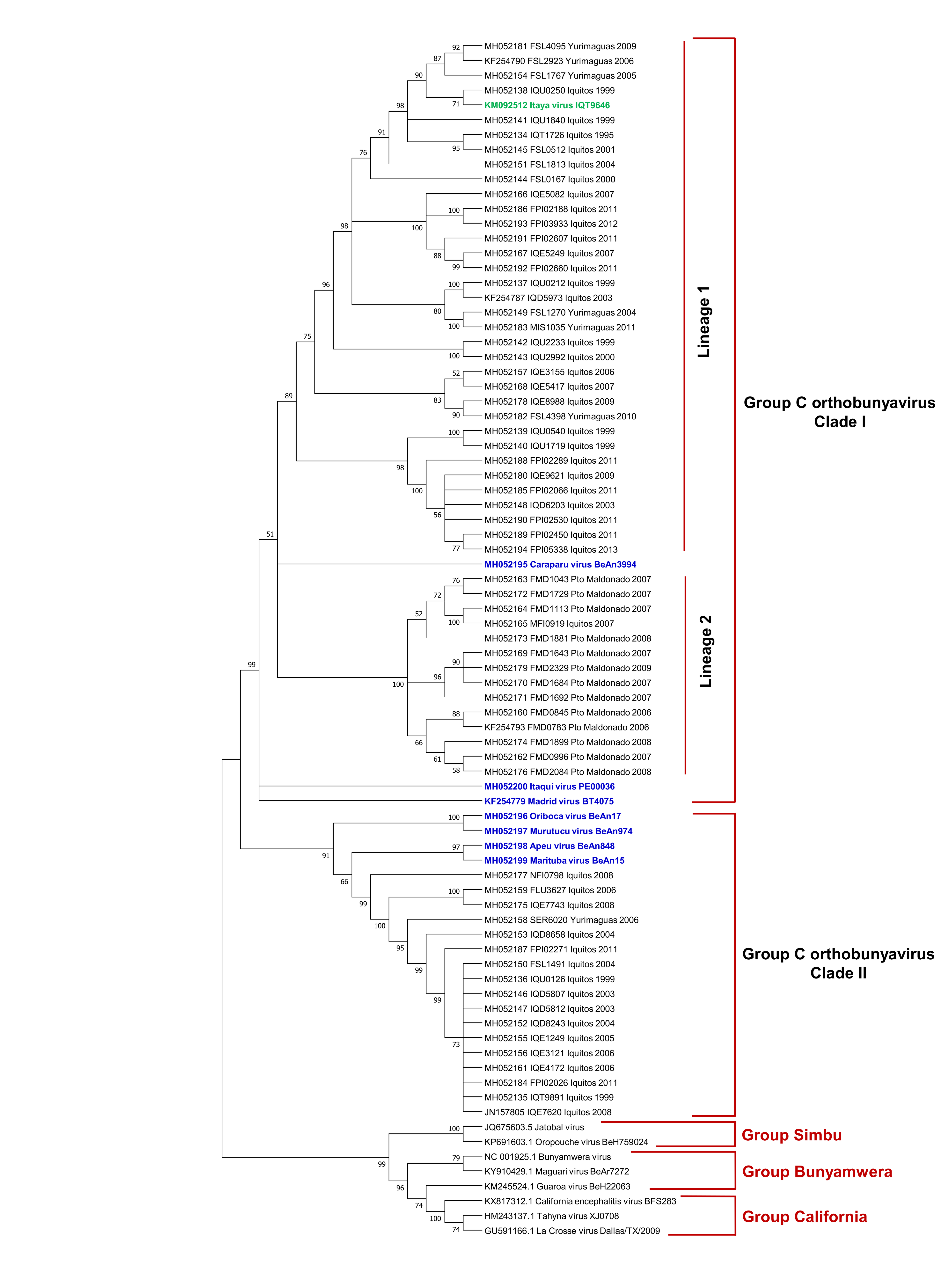

Supplement: S6 Fig — The MP tree was constructed using the Maximum Parsimony method. Bootstrap consensus tree inferred from 500 replicates is taken to represent the evolutionary history of the taxa analyzed on the bases of partial coding sequence of RdRp. The MP tree was obtained using the Subtree-Pruning-Regrafting (SPR) algorithm. Members of the Simbu, California and Bunyamwera serogroups were used as outgroups to root the tree. (TIF) [file pone.0200576.s007.tif]
